# Our Experience in Tracking the Tract: Normal Biliary Anatomy and Variants on Magnetic Resonance Cholangiopancreatography in Living Donor Liver Transplantation

**DOI:** 10.7759/cureus.34695

**Published:** 2023-02-06

**Authors:** Sriram Jaganathan, Brijesh Ray, Jyotirmayi Velaga

**Affiliations:** 1 Radiology, Global Hospitals, Bengaluru, IND; 2 Radiology, Aster Medcity, Kochi, IND; 3 Radiology, Global Hospitals, Chennai, IND

**Keywords:** living donor liver transplant (ldlt), living donor liver transplantation, cholangiopancreatography, magnetic resonance, liver transplantation, living donor, variations, biliary anatomy

## Abstract

Objective

Biliary anatomy is of paramount importance for hepatobiliary pancreatic surgeons for operative planning. Preoperative assessment with magnetic resonance cholangiopancreatography (MRCP) to evaluate the biliary anatomy plays a vital role, especially for prospective liver donors in living donor liver transplantation (LDLT). Our objective was to evaluate the diagnostic accuracy of MRCP in assessing the anatomical variations of the biliary system and the frequency of biliary variation in the donors of LDLT.

Materials and Methods

Sixty-five donors of living donor liver transplantation in the age range of 20 to 51 years were studied retrospectively to evaluate the anatomical variations of the biliary tree. As a part of the pre-transplantation donor workup, MRI with MRCP was performed in a 1.5T machine for all these candidates. MRCP source data sets were processed with maximum intensity projections, surface shading, and multi-planar reconstructions. Images were reviewed by two radiologists, and the classification system of Huang et al. was utilized to evaluate the biliary anatomy. The results were compared with the intraoperative cholangiogram, considered the gold standard.

Results

We identified standard biliary anatomy in 34 candidates (52.3%), and variant biliary anatomy was observed in 31 candidates (47.7%) on MRCP. An intraoperative cholangiogram showed standard anatomy in 36 candidates (55.4%) and biliary variation in 29 candidates (44.6%). Our study showed a sensitivity of 100% and a specificity of 94.5% for identifying biliary variant anatomy on MRCP in comparison with the gold standard intraoperative cholangiogram. The accuracy of MRCP in detecting the variant biliary anatomy in our study was 96.9%. The most common biliary variation was the right posterior sectoral duct draining into the left hepatic duct, Huang type A3.

Conclusion

The frequency of biliary variations is high in potential liver donors. MRCP is sensitive and highly accurate in identifying the biliary variations of surgical significance.

## Introduction

Living donor liver transplantation (LDLT) is an alternative therapeutic option to cadaveric liver transplantation for end-stage liver disease patients. Imaging plays a crucial role in assessing the recipients and the living donors prior to the transplantation as well as in the postoperative period for complications. Biliary complications are one of the major causes of morbidity in these living donor liver transplantations. Preoperative evaluation of prospective liver donors for variants in the biliary anatomy is the key to minimizing major biliary complications following surgery [1-4]. Magnetic resonance cholangiopancreatography (MRCP) is the non-invasive imaging modality of choice to evaluate the biliary system and pancreatic duct. In recent days, MRCP has gained wide acceptance as the most reliable alternate modality to intraoperative cholangiography for clearly demonstrating the biliary system [5-7]. Our objective was to evaluate the diagnostic accuracy of MRCP in assessing the anatomical variations in the biliary system and the frequency of biliary variation in liver donors.

## Materials and methods

A retrospective study was done utilizing the preoperative planning magnetic resonance cholangiopancreatography (MRCP) performed for living donor liver transplantation (LDLT). Sixty-five donor MRCP examinations performed for LDLT between January 2013 and July 2018 at the Global Hospitals Group (Chennai and Bengaluru, India) and Aster Medcity (Kochi, India) were studied retrospectively to evaluate the frequency of anatomical variations of the biliary tree. The age group of the donors was in the range of 20 to 51 years. A total of 105 MRCP studies performed for potential liver donors during the period were evaluated. We excluded 25 MRCP studies as there were no intraoperative cholangiograms available for us to compare, and we also excluded an additional 15 MRCP studies that had respiratory movement artifacts that prevented us from having a meaningful interpretation. The remaining 65 MRCP examinations were included in the study.

Inclusion and Exclusion Criteria

All the completed MRCP studies, without significant respiratory movement artifacts, performed as a part of the preoperative planning protocol for LDLT were included in the study. Donors who were not able to complete the study or whose images contained respiratory movement artifacts impeding accurate interpretation were excluded. We also excluded donors if the intraoperative cholangiogram images were not made available for us to compare.

The institutional review board approved the retrospective study. Informed consent was waived.

As a part of the pre-transplantation donor workup, MRI with MRCP was performed on a 1.5T MRI GE machine for all these candidates. A single-shot fast spin echo sequence (SSFSE, GE Medical Systems) was performed for all the donors after the planning sequences. A respiratory-gated three-dimensional turbo spin echo sequence was also performed that offered a high signal-to-noise ratio, improved spatial resolution, and high contrast-to-noise ratio. Each donor received a phased-array body coil. We preferred to do the procedure after an overnight fast. Sometimes, we also gave pineapple juice to the donors as an oral negative contrast for a better appreciation of the biliary tract.

MRCP source data sets were processed with maximum intensity projections, surface shading, and multi-planar reconstructions. The images were reviewed by two radiologists and compared with an intraoperative cholangiogram. Intraoperative cholangiograms performed via cystic duct cannulation were considered the gold standard. Sensitivity, specificity, and accuracy were calculated based on the findings.

## Results

The study included 65 liver donors ranging in age from 20 to 51 years, with an average age of 38 years. There were 41 men and 24 women. Thirty-four candidates (52.3%) had standard biliary anatomy on magnetic resonance cholangiopancreatography (MRCP). Biliary tract variant anatomy was observed in 31 candidates (47.7%) on MRCP (Table 1). An intraoperative cholangiogram showed normal anatomy in 36 candidates (55.4%) and biliary variation in 29 donors (44.6%) (Table 1). The additional two uncommon segmental duct variants identified on MRCP were found to have standard anatomy on operative cholangiogram, probably due to artifactual misinterpretation of MRCP. Though we used the Huang classification system as the basis (Table 2), we described the biliary anatomy based on the sectoral duct drainage, as this was preferred by our hepatobiliary surgeons.

**Table 1:** Standard anatomy and biliary variation as seen on MRCP and intraoperative cholangiogram.

Biliary anatomy	Number	Percentage
MRCP		
Standard conventional	34	52.3%
Variant	31	47.7%
Intraoperative cholangiogram		
Standard conventional	36	55.4%
Variant	29	44.6%

**Table 2:** Huang's classification of biliary system anatomy RPSD: right posterior sectoral duct; RASD: right anterior sectoral duct; LHD: left hepatic duct; CHD: common hepatic duct

Huang classification types	
A1	The right posterior sectoral duct (RPSD) drains into the right anterior sectoral duct (RASD)
A2	Trifurcation pattern of insertion of RPSD, RASD, and the left hepatic duct (LHD)
A3	RPSD drains into the LHD
A4	RPSD drains into the common hepatic duct (CHD)
A5	RPSD drains into the cystic duct

**Figure 1:**
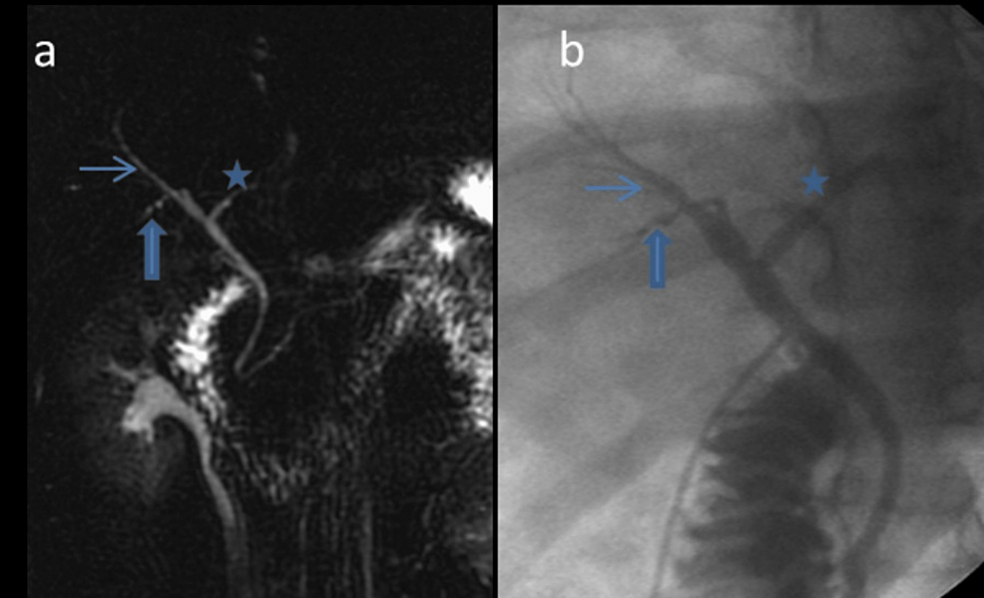
MRCP showing standard biliary anatomy (Huang A1). (a) The right anterior (thin arrow) and right posterior sectoral ducts (bold arrow) join to form the right hepatic duct. The right hepatic duct and the left hepatic duct (asterisk) join to form the common hepatic duct; (b) a preoperative cholangiogram of the same patient during transplantation showed a standard branching pattern.

**Figure 2:**
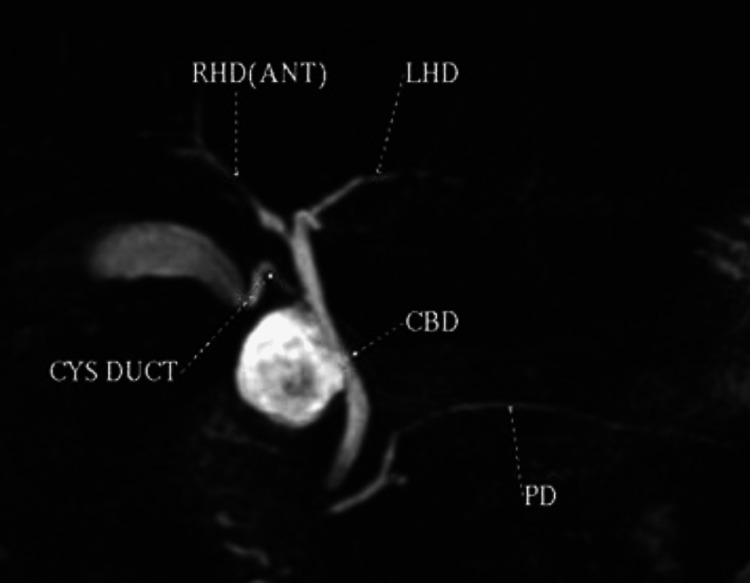
Standard anatomy. The cystic duct drains normally into the common hepatic duct, forming the common bile duct. RHD: right hepatic duct; LHD: left hepatic duct; CYS DUCT: cystic duct; CBD: common bile duct; PD: pancreatic duct

The standard biliary anatomy (Figures 1-2) is where the right anterior sectoral duct and the right posterior sectoral duct unite to form the right hepatic duct. The right hepatic duct joins the left hepatic duct to form the common hepatic duct. The cystic duct joins the common hepatic duct to form the common bile duct.

**Figure 3:**
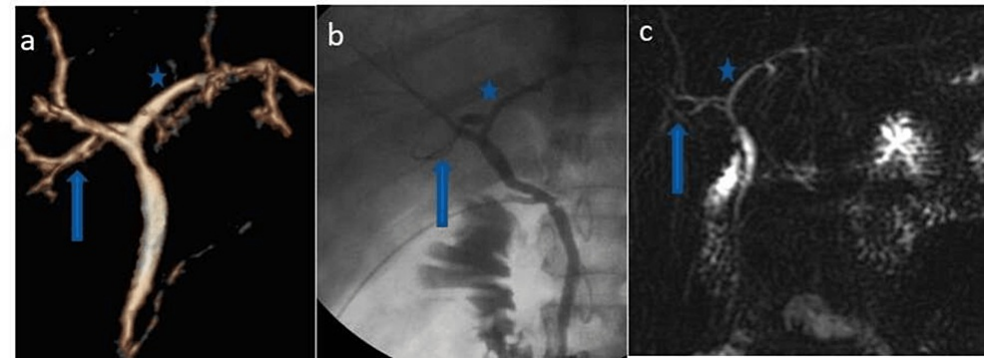
The right posterior sectoral duct (arrows) drains into the left hepatic duct (asterisk). Volume-rendered images (a), intraoperative cholangiogram (b), and MRCP images (c) show the most common variation (Huang A3).

**Figure 4:**
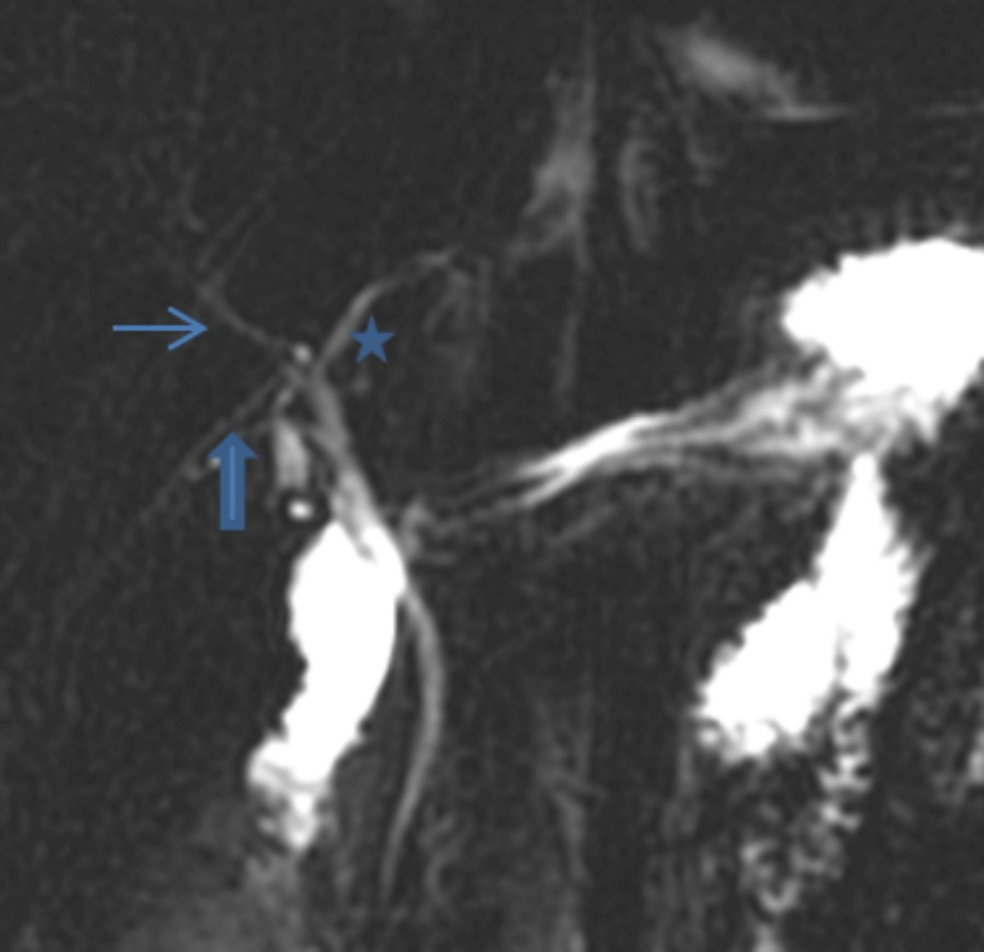
Trifurcation pattern: the right anterior sectoral duct (thin arrow), the right posterior sectoral duct (bold arrow), and the left hepatic duct (asterisk) join to form the common hepatic duct (Huang A2).

**Figure 5:**
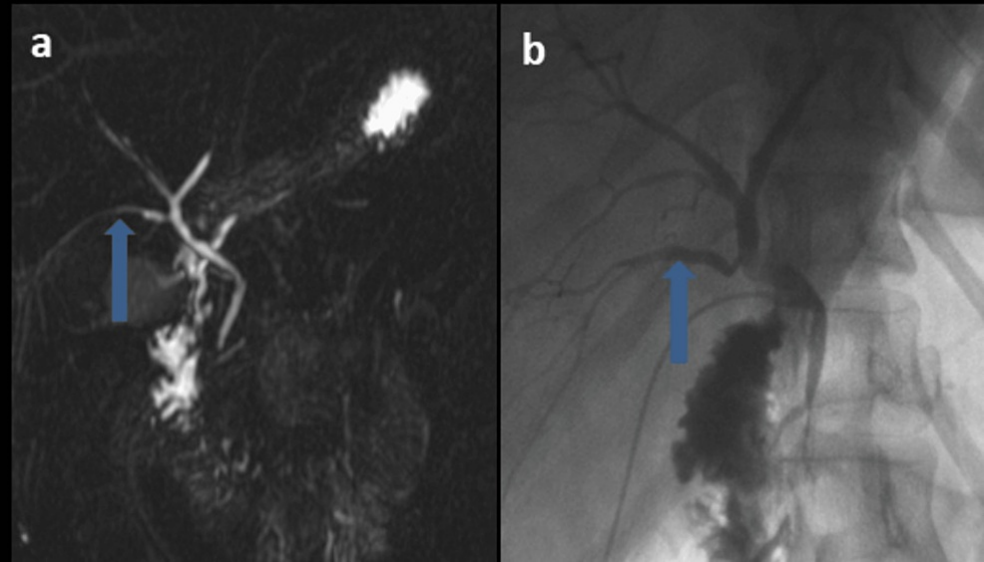
The right posterior sectoral duct (arrows) drains into the common hepatic duct: MRCP (a) and intra-operative cholangiogram (b) (Huang A4).

The most common biliary tree variation was the drainage of the right posterior sectoral duct into the left hepatic duct, which was observed in 16 candidates (24.6%) (Figure 3). A trifurcation biliary pattern was seen in six candidates (9.2%) (Figure 4). The right posterior sectoral duct joined the common hepatic duct in four candidates (6.2%) (Figure 5).

**Figure 6:**
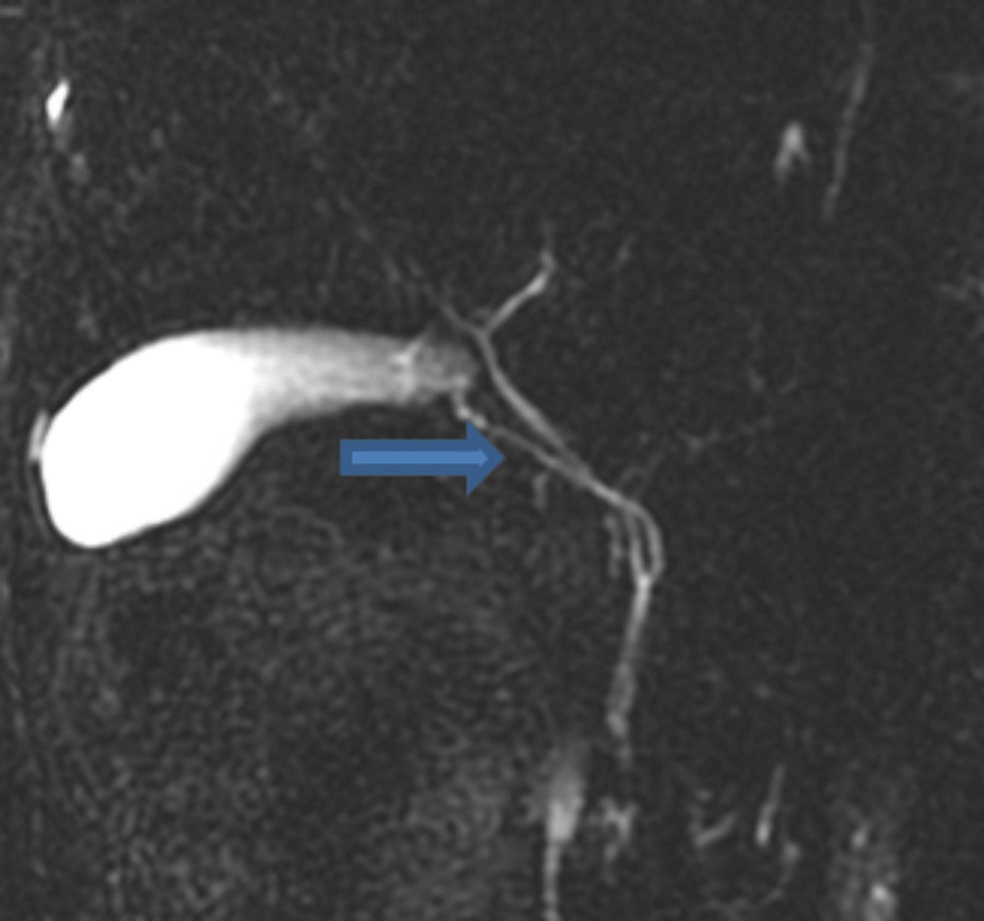
Low insertion of the cystic duct (arrow)

**Figure 7:**
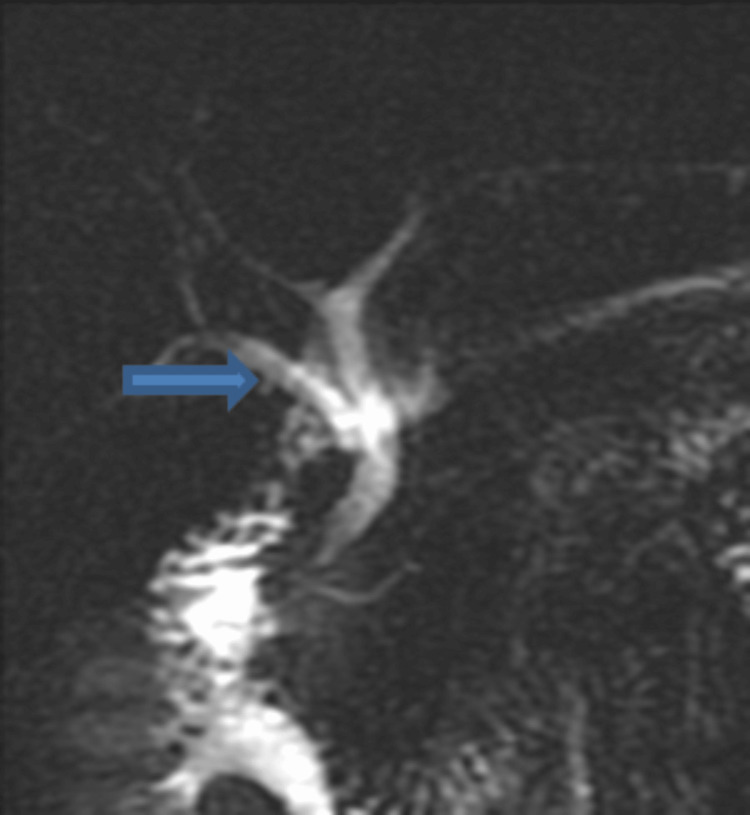
The right posterior sectoral duct (arrow) drains into the common bile duct

Other uncommon biliary variations encountered were the low insertion of a long cystic duct medially (Figure 6), a right posterior sectoral duct draining into the common bile duct (Figure 7), and unusual segmental duct variations.

The variants are summarized in Table 3 and Table 4.

**Table 3:** Biliary variant anatomy as seen on preoperative MRCP RPSD: right posterior sectoral duct; CBD: common bile duct

	Number	Percentage
Variant biliary anatomy		
RPSD to the left hepatic duct	16	24.6%
Trifurcation	6	9.2%
RPSD to the common hepatic duct	4	6.2%
RPSD to CBD	1	1.5%
Cystic duct variations	1	1.5%
Other uncommon variations	3	4.7%

**Table 4:** Biliary variant anatomy as seen on intraoperative cholangiogram RPSD: right posterior sectoral duct; CBD: common bile duct

	Number	Percentage
Variant biliary anatomy		
RPSD to the left hepatic duct	16	24.6%
Trifurcation	6	9.2%
RPSD to the common hepatic duct	4	6.3%
RPSD to CBD	1	1.5%
Cystic duct variations	1	1.5%
Other uncommon variations	1	1.5%

Our study showed a sensitivity of 100% and a specificity of 94.5% for identifying biliary variant anatomy on MRCP in comparison with the gold standard intraoperative cholangiogram. We did not find any left hepatic duct variations. The accuracy of the MRCP in detecting the variant biliary anatomy in our study was 96.9%.

## Discussion

Liver transplantation is the treatment of choice for irreversible acute and chronic liver disease. The types of transplantation are cadaveric and living donor liver transplantation (LDLT). Though cadaveric transplantation is technically highly successful, organ availability is limited. The main advantage of living donor liver transplantation is the ability to perform the transplant on an elective basis. Imaging plays a key role in assessing the recipients and the living donors prior to the transplant as well as in the postoperative period for complications.

Recognizing the portal venous, hepatic venous, hepatic arterial, and biliary anatomy is essential to liver resection and associated vascular and biliary reconstructions [8-10].

Normal biliary anatomy is parallel to the portal venous supply, with the right hepatic duct draining segments V- VIII and the left hepatic duct draining segments II-IV [10]. The right anterior sectoral duct drains segments V and VIII. The right posterior sectoral duct drains segments VI and VII. It is important to be aware that the right posterior sectoral duct has a more horizontal course and the right anterior sectoral duct has a vertical course. The right anterior sectoral duct and the right posterior sectoral duct unite to form the right hepatic duct. The right hepatic duct joins the left hepatic duct to form the common hepatic duct. The duct draining the caudate lobe joins either the right or the left hepatic duct [11]. The cystic duct joins the common hepatic duct and forms the common bile duct. Puente et al., in their 3845 intra-operative cholangiograms, found this standard biliary anatomy in almost 58% of the population [11]. In our study, 34 candidates (52.3%) had this standard biliary anatomy.

Biliary complications, occurring in 3%-10% of donors, represent the most common cause of morbidity in living donor liver transplantation. The complications include biliomas secondary to bile leakage and bile duct strictures. Bile leakage may occur at the biliary anastomosis and rarely along the liver parenchymal transection [12-14]. It has been demonstrated that detailed preoperative evaluation and understanding of the biliary anatomic variants are useful to prevent biliary complications, helping surgeons safely perform donor hepatectomy [13-15].

Our study results correlate with the magnetic resonance cholangiopancreatography (MRCP) studies on potential liver donors by Wang et al. Their study on 62 liver donors showed a 56% standard biliary anatomical pattern [16]. In our study, we had 34 out of 65 candidates with standard biliary anatomy, representing 52.3%. Basaran et al. evaluated 40 potential liver donors and found standard biliary anatomy in 67.5% of donors [17]. Naeem et al., in their study of 369 patients, identified conventional standard anatomy in 65.8% [18]. Higher numbers for standard biliary anatomy were seen in the studies by Basasran et al. and Naeem et al. compared to our study.

The most common variation observed in our study was the drainage of the right posterior sectoral duct into the left hepatic duct in 16 out of 65 candidates (24.6%). This was the most common variant of anatomy in the studies of Basaran et al. (20%) and Wang et al. (18%) [16,17].

The second common variation in our study was the trifurcation pattern, which was seen in six candidates, constituting 9.2%. This variation was seen in 5% of donors in Basaran et al. and 11% of their donors in Wang et al. [16,17].

The next common variation in our study, the right posterior sectoral duct draining into the common hepatic duct, was observed in 6.2% of candidates, which was seen in 8% in Wang et al. and 2.5% in Basaran et al. [16,17].

Other uncommon variations seen in our study include the right posterior duct draining into the common bile duct, one of the segment four ducts draining into the right hepatic duct, and other uncommon segmental duct variations. There are multiple other uncommon variations described by Nayman et al. [5].

Hariri and Riad, in their study on 120 subjects, demonstrated standard anatomy (Huang A1) in 65.83% and the most common biliary variation, Huang A3, in 13.3% [19]. A higher percentage of standard anatomy was found compared to our study. However, the most common biliary variation, Huang A3, was similar to our study.

Sarawagi et al., in their evaluation of 224 MRCP patients, found a similar prevalence of biliary anatomy, with standard anatomy in 55.3% of the patients and variant anatomy in 44.7%. The most common variation was the right posterior duct draining into the left hepatic duct (27.6%) [20]. Our study results also correlate with the results of Sarawagi et al.

Limitations

Our study has certain limitations. A limited number of subjects were evaluated, which could be one of the reasons for the higher frequency of biliary variation. We found very few segmental duct variations and no left hepatic duct variations, which could be attributed to the small sample size. This limitation can be overcome by a larger study.

## Conclusions

Variations in the intrahepatic and extrahepatic biliary anatomy are multiple, including a few common and many uncommon variants. Biliary complications are one of the major causes of morbidity in living donor liver transplantation. MRCP is an excellent tool for preoperative donor evaluation for LDLT and is highly accurate in identifying biliary tree variations. We observed a high frequency of biliary variations in candidates for donor evaluation that were surgically significant. It is imperative for surgeons to be aware of the variant anatomy before donor hepatectomy to avoid biliary complications.
